# Giardiavirus rewires host translation and glycolytic metabolism to support its replication in *Giardia duodenalis*

**DOI:** 10.1080/21505594.2025.2605746

**Published:** 2025-12-24

**Authors:** Lu Li, Lili Cao, Chongli Zhong, Nan Zhang, Xin Li, Xiaocen Wang, Yanbing Guo, Yanhui Yu, Jianhua Li, Xichen Zhang, Yukun Le, Jianqi Yuan, Pengtao Gong

**Affiliations:** a State Key Laboratory for Diagnosis and Treatment of Severe Zoonotic Infectious Diseases, Key Laboratory for Zoonosis Research of the Ministry of Education, Institute of Zoonosis, and College of Veterinary Medicine, Jilin University, Changchun, China; b Laboratory of Parasitology, Jilin Academy of Animal Husbandry and Veterinary Medicine, Changchun, China; c Department of Endocrinology, The Second Norman Bethune Hospital of Jilin University, Changchun, China

**Keywords:** *Giardia duodenalis*, Giardiavirus, translation efficiency, energy metabolic, glycolysis, enolase

## Abstract

*Giardia duodenalis* is an intestinal protozoan parasite responsible for giardiasis, a disease primarily characterized by diarrhea and associated with long-term complications such as malnutrition and growth impairment in children. The presence of Giardiavirus (GLV) has been shown to attenuate pathological damage in *G. duodenalis*-infected murine models and modulate distinct pro-inflammatory responses in host cells stimulated by *Giardia*. However, the understanding of the impact of the GLV on the *G. duodenalis* itself remains limited. Here, we found that GLV infection interfered with the host protein expression system by reducing both mRNA and protein levels of *Giardia* genes, while paradoxically enhancing mRNA translation efficiency. Additionally, GLV infection induced energy metabolic reprogramming in *Giardia*, as evidenced by the identification of 21 significantly altered energy metabolites. KEGG enrichment analysis revealed glycolysis/gluconeogenesis as the most prominently enriched metabolic pathway in GLV-infected *Giardia*. Notably, glycolysis continued to be upregulated with successive passages of GLV infection, even after the GLV load plateaued. The glycolytic enzyme enolase was found to be closely associated with GLV infection within *Giardia*, and morpholino-mediated knockdown of enolase expression resulted in a significant reduction in GLV replication. Overall, these findings demonstrate that GLV infection manipulates host translation and energy metabolic pathways to facilitate its persistence in *G. duodenalis*, and reveal both GLV and host metabolic targets as promising research subjects for developing drugs and vaccines for the prevention and treatment of giardiasis.

## Introduction

*Giardia duodenalis* is a zoonotic protozoan parasite that causes giardiasis, a diarrheal disease of public health concern, affecting a wide range of mammalian hosts, including humans. A recent systematic review, which encompassed 861 studies across 89 countries and 4.9 million animals, estimated the global pooled prevalence of *Giardia* infection in nonhuman mammals at 13.6% [1]. Studies have shown that *G. duodenalis* infection is an independent risk factor for impaired early-life linear growth in children from low- and middle-income countries (LMICs) [2–4]. Recently, a survey conducted by Bele Gesgar Hospital in Oromia Region, Ethiopia, on 134 children with intestinal parasitic infections (IPIs) found that the prevalence of *G. duodenalis* was 9.48% [5]. Tamomh et al. also found that in the IPIs survey in Kosti City, White Nile State, Sudan, the prevalence of *G. duodenalis* among the children was 25.0% [6].

Giardiavirus (GLV) is a non-segmented double-stranded RNA virus that belongs to the *Totiviridae* family. It is also the only virus identified within the genus Giardiavirus, characterized by an icosahedral, non-enveloped structure with an approximately 6.3 kb genome, encoding the capsid protein (ORF1) and the RNA-dependent RNA polymerase (ORF2) [7]. Previous studies have reported that up to 30% of the *G. duodenalis* isolates tested were infected with GLV [8,9]. This prevalence underscores the significance of understanding GLV’s impact on *G. duodenalis*, as it may influence the parasite’s biology and host interactions. Our previous research found that GLV infection can alleviate the progression of disease in mice caused by the *Giardia* GS isolate, indicating a potential modulatory effect of the GLV on the host-parasite dynamics [10]. Pu and Li et al. reported that GLV infection can induce differential pro-inflammatory cytokine secretion in various cell lines infected with *G. duodenalis*, suggesting that GLV infection may modulate *Giardia*-induced host immune responses [11,12]. Understanding the influence of GLV on *Giardia* is crucial for comprehending the pivotal role of GLV in regulating the biology and virulence of *Giardia*.

As obligate intracellular, non-cellular organisms, viruses hijack host cellular machinery – such as proteins and metabolic pathways – to produce their components and replicate [13,14]. Viral infection can significantly remodel the host proteome, affecting protein expression levels and post-translational modifications [15]. For example, Leishmaniavirus 1 (LRV1) can regulate the upregulation of gene expression and increase translation efficiency in *Leishmania* [16–18]. Trichomonas vaginalis virus infection leads to the protein expression variations in *Trichomonas vaginalis* [19]. Furthermore, viruses also alter host metabolic networks to provide essential resources and energy for virus replication through the control of important metabolic processes, such as glycolysis, fatty acid synthesis, and amino acid metabolism [20,21]. This metabolic reprogramming not only affects the replication efficiency of the virus but may also impact the outcome of viral infections, such as host’s immune response and disease progression [22]. Hepatitis C virus (HCV) and Zika virus (ZIKV) profoundly remodel host lipid metabolism, promoting lipid synthesis to support viral particle formation and potentially aiding immune evasion [23,24]. Dengue virus (DENV) infection markedly alters host glycolytic metabolism by increasing glucose consumption and upregulating glycolysis-related enzymes to meet the energetic and biosynthetic demands of viral replication [25]. However, how Giardiavirus (GLV) regulates host translation and impacts energy metabolism in *Giardia duodenalis* remains unclear.

In this study, we investigated the effects of GLV on the mRNA and protein levels of *G. duodenalis*, as well as its impact on the parasite’s energy metabolism. Our findings suggest that GLV facilitates its own replication by hijacking host protein synthesis and reprogramming energy metabolism.These findings underscore the importance of understanding GLV-*Giardia* interactions and provide a novel theoretical framework for the development of drugs and vaccines aimed at preventing and treating giardiasis.

## Material and methods

### Parasites

The *G. duodenalis* trophozoites used in this study included both GLV-free and GLV-infected strains. The GLV-free strain, designated WB^−GLV^, was derived from the *G. duodenalis* WB isolate, Assemblage A1 (ATCC30957; American Type Culture Collection, Manassas, VA, USA). GLV-positive trophozoites, all belonging to Assemblage AI, were obtained from the Parasite Laboratory of the College of Veterinary Medicine, Jilin University. These trophozoites included a naturally infected strain (*Giardia*^+GLV^) and a laboratory-generated strain (WB^+GLV^, acquired by introducing Giardiavirus into WB^−GLV^ trophozoites) [10]. *G. duodenalis* trophozoites were cultured at 37°C in modified TYI-S-33 medium supplemented with 12.5% heat-inactivated fetal bovine serum (Every Green, Zhejiang), 0.1% bovine bile (Sigma, USA), 50 µg/mL gentamicin sulfate, 100 U/mL penicillin and 100 U/mL streptomycin (Biological Industries, Israel). After cooling the tubes on ice for 20 min, trophozoites were harvested by centrifugation at 400 g for 8 min at 4°C.

### qPCR

An equal number of *Giardia* trophozoites (including WB^−GLV^, *Giardia*^+GLV^ and WB^+GLV^ strains) used in this study were harvested during the logarithmic growth phase. Total RNA was isolated using TRIzol reagent (TransGen Biotech, China). DNA-free RNA (2 µg) was reverse transcribed into cDNA using the All-In-One 5× RT MasterMix (Abmgood, Canada) according to the manufacturer’s protocol. qPCR was carried out on an AriaMx Real-Time PCR System (Agilent Technologies, USA) using gene-specific primers and BlasTaq™ 2× qPCR MasterMix (SYBR Green, Abmgood) to assess mRNA expression levels. Primer sequences for the 30 genes are shown in Supplementary Table 1. Each qPCR reaction (20 μL total volume) contained 10 μL of 2× qPCR MasterMix, 0.2 μM of each primer, 1 μL of 10-fold diluted cDNA, and nuclease-free water. The amplification protocol included an initial denaturation at 95 °C for 30 s, followed by 40 cycles of 95 °C for 10 s and 60 °C for 30 s with real-time fluorescence detection. A melting curve analysis was performed at the end of the run. Relative RNA expression levels were calculated using the 2^–ΔΔCt method, with *G. duodenalis* 18S rRNA and actin genes as internal reference genes for normalization.

### Western blot assays

*Giardia* trophozoites were collected and lysed in RIPA buffer (Solarbio, China) containing 1% (v/v) 100×protease inhibitor cocktail, followed by ultrasonic lysis. The lysates were centrifuged, and protein concentrations were determined using a BCA protein assay kit. Samples were then mixed with 6× loading buffer (TransGen, China) and boiled for 10 min. Protein molecular weight markers (G2091 and G2083, Servicebio, China; DM111-02, TransGen) were used to estimate the molecular weights of the target protein. Equal amounts of protein (30 μg/well) were separated on 12% Tris – glycine gels (Epizyme, China) or 4–20% Bis – Tris gradient precast gels (Absin, China) and transferred to 0.22 μm PVDF membranes (Merck Millipore). Membranes were blocked with protein-free rapid blocking buffer (Epizyme) for 30 min at room temperature (RT), and then incubated overnight at 4 °C with primary antibodies against Rab2a, Enolase, Capsid, Tubulin (1:500–1:1000) and HA (1:5000; Proteintech, China). Polyclonal antibodies against Capsid and Tubulin were provided by Zhao [26]. The anti-Rab2a [10] and anti-Enolase polyclonal antibodies were developed and stored in our laboratory. After washing with PBST, membranes were incubated at RT for 1 h with HRP-conjugated goat anti-rabbit/anti-mouse IgG (1:5000, Proteintech), and signals were visualized using the Omni-ECL™ Pico Light Chemiluminescence Kit (Epizyme) on the ChemiCapturePAD imaging system (Clinx, China).

### Polysome profiling and RNA-seq analyses

Polysome profiling is a powerful tool for assessing the translational status of mRNAs by quantifying monosomes (inefficient translation) and polysomes (efficient translation), and a higher polysome-to-monosome (P/M) ratio indicates greater ribosomal loading on mRNAs, reflecting enhanced translation efficiency [27,28]. We followed a previously described protocol with a few modifications [29]. *Giardia* trophozoites were snap-frozen in liquid nitrogen. For polysome and monosome RNA isolation, 10^^8^ frozen trophozoites were lysed in 500 μL of polysome extraction buffer containing 25 mM Tris-HCl (pH 7.4), 150 mM NaCl, 5 mM MgCl_2_, 1 mM dithiothreitol, 1 mM phenylmethanesulfonyl fluoride, 100 μg/mL cycloheximide, 400 U/mL RNasin, 1% deoxycholic acid, and 0.5% (v/v) NP-40. After incubation on ice for 5 min with gentle inversion, cellular debris was pelleted by centrifugation at 13,000 g for 10 min at 4°C. 100 μL of trophozoites extract was then carefully layered onto a 10%–45% sucrose gradient, and ultracentrifuged at 36,000 rpm for 3 h at 4°C using a Beckman Optima XE-100 ultracentrifuge (Beckman, Germany) equipped with an SW 40 Ti swinging-bucket rotor. This process facilitated the separation of monosome and polysome RNAs based on their sedimentation coefficients along the sucrose gradient. Eighteen fractions were collected using a density gradient fractionator (Biocomp, CAN) and further divided into mixed monosome fractions (fractions 5–12) and mixed polysome fractions (fractions 13–18). The translation efficiency was quantified by calculating the ratio of the area under the curve (AUC) for polysome fractions and monosome fractions. mRNA extracted from the monosome or polysome fractions was used to construct sequencing libraries with the TruSeq PE Cluster Kit v3-cBot-HS (Illumina). These libraries were subjected to high-throughput sequencing on a HiSeq-PE150 platform (Illumina) at Novogene Bioinformatics Technology (China).

### Metabolite profiling

WB^+GLV^, WB^−GLV^ and *Giardia*^+GLV^ trophozoites were snap-frozen on dry ice and submitted to MetWare company (Wuhan, China) for metabolomic analysis. Briefly, approximately 50 mg of trophozoites was mixed with 500 μL of 70 % methanol, vortexed for 3 min at 2500 rpm, and centrifuged at 12,000 rpm for 10 min at 4 °C. 300 μL of the supernatant was collected and incubated at −20°C for 30 min, followed by centrifugation again at 12,000 rpm for 10 min at 4°C. 200 μL of the supernatant was transferred through a protein precipitation plate. Metabolite profiling was performed by MetWare using an AB Sciex QTRAP 6500 LC-MS/MS platform. Energy metabolites were quantified using scheduled multiple reaction monitoring (MRM). Metabolite data acquisition was conducted using Analyst software version 1.6.3 (Sciex), and quantification was performed with MultiQuant software version 3.0.3 (Sciex). Mass spectrometer parameters, including declustering potentials (DP) and collision energies (CE), were individually optimized for each MRM transition. A targeted set of MRM transitions was monitored within specific retention time windows, based on the elution profiles of the metabolites.

### Glycolytic stress assay test

The metabolic profile of *Giardia* trophozoites was determined using a Seahorse XF24 Extracellular Flux Analyzer (Seahorse Biosciences, USA) [30]. The XF24 Cell Culture Microplates (Agilent, USA) were pretreated with the cell and tissue adhesive (Corning, USA) for 30 min. Then, the WB^+GLV^ trophozoites (cultured for 5 and 40 generations) were used as experimental groups, WB^−GLV^ and *Giardia*^+GLV^ trophozoites served as negative and positive controls, respectively. A total of 8 × 10^5^ trophozoites were seeded into each well of a seahorse XF24 cell culture microplate. After a 30 min attachment period at 37°C, the culture medium was replaced with Seahorse XF DMEM medium (containing 5 mM HEPES and devoid of phenol red, sodium bicarbonate, glucose, L-glutamine, and sodium pyruvate). The trophozoites were then incubated for an additional 30 min prior to analysis. Metabolic measurements were subsequently performed using the Seahorse XF24 Extracellular Flux Analyzer. Glycolytic activity was evaluated using the Seahorse XF Glycolytic Stress Test Kit (Agilent, Santa Clara, CA; 103,020-100), following the manufacturer’s protocols. The levels of glycolysis, glycolytic capacity, and glycolytic reserve were monitored.

### Enolase enzymatic activity

Enolase activity of *Giardia* lysates was measured using a direct spectrophotometric assay by monitoring the conversion of 2-phosphoglycerate (2-PGA) to phosphoenolpyruvate (PEP) through absorbance at 240 nm. PEP exhibits a strong specific absorbance peak at 240 nm, while 2-PGA shows minimal absorbance at this wavelength [31]. The reaction was conducted in a 96-well plate format with the direct assay performed in UV-transmissible plates. The reaction was initiated by adding 2.5 mM 2-PGA, 1.5 mM MgCl_2_, and 50 mM Tris-HCl (pH 7.5). Optical density (OD) at 240 nm was recorded during the reaction [32].

### Construction of Giardia expressing the hemagglutinin (HA) epitope-tagged Gd-enolase proteins

A partial 820-bp fragment of the *Giardia* enolase (Gd-enolase) gene (*GL50803_0011118*) was amplified from *Giardia* genomic DNA by PCR using the primers (Supplementary Table .2). The plasmid pKS-3 HA.neo was kindly provided by Professor Paredez (Department of Biology, University of Washington). Next, the plasmid pKS-3 HA.neo was digested with restriction enzymes *NotI* and *SalI*. The amplified enolase gene fragment was ligated into the double-digested pKS-3 HA.neo vector to generate the recombinant plasmid pKS-enolase.neo containing an HA tag. Positive clones were screened by colony PCR, and the construct was confirmed by 1% agarose gel and DNA sequencing (Sangon Biotech, China). Subsequently, the recombinant plasmid HA-tagged pKS-enolase.neo was linearized by inverse PCR [33]. An aliquot (15 ~ 20 μg) of linearized HA-tagged pKS-enolase.neo plasmid was transfected into 1 × 10^7^ WB^+GLV^ trophozoites via electroporation (Bio-Rad, USA) under the following conditions: 350 V, 1000 μF and 700 Ω. Expression of HA-tagged Gd-enolase was confirmed by Western blotting and immunofluorescence microscopy. *Giardia* trophozoites transfected with the empty vector pKS-3 HA.neo served as negative controls.

### Immunofluorescence assay

L-lysine-coated glass slides (Corning, USA) were placed in 24-well plates, and 5 × 10^5^ *Giardia* trophozoites were added to each well. The plates were incubated at 37 °C for 30 min to allow *Giardia* adhesion. After incubation, non-adherent cells were gently removed by washing with PBS. The adherent *Giardia* were then fixed with 4% paraformaldehyde for 20 min, and permeabilized with 0.25% Triton X-100 for 10 min. After blocking with 3% bovine serum albumin (BSA) for 30 min, trophozoites were incubated overnight at 4°Cwith primary antibodies (rabbit anti-HA tag polyclonal antibody, 1:100; Proteintech, China), followed by incubation with fluorophore-conjugated secondary antibody (Alexa Fluor 488-conjugated goat anti-rabbit IgG,1:200; Proteintech, China) at RT for 1 h. Samples were mounted with antifade mounting medium containing DAPI (Beyotime, China) and imaged using an inverted confocal microscope (FV 3000; Olympus, Japan).

### Morpholino knockdown

Specific morpholino oligonucleotides targeting Gd-enolase, along with a nonspecific control morpholino, were custom-designed and synthesized by Gene Tools (Philomath, OR, USA)(Supplementary Table 3). *G. duodenalis* WB^+GLV^ (5 × 10^6^ trophozoites in 0.3 mL medium) were treated with lyophilized morpholino at a final concentration of 100 nM prior to electroporation [34]. At 24 h post-transfection, Gd-enolase expression in trophozoites was evaluated by Western blotting and immunofluorescence using anti-HA antibodies.

### Statistical analysis

Statistical analyses were conducted using unpaired Student’s t-tests (one- or two-tailed) or one-way ANOVA, as appropriate. Data are presented as the mean ± standard error of the mean (SEM). Statistical significance was defined as *p ≤ 0.05, **p ≤ 0.01, and ***p ≤ 0.001. All analyses were performed using GraphPad Prism software. The results shown are representative of at least three independent experiments with consistent outcomes.

## Results

### Giardiavirus infection regulates mRNA and protein abundance in G. duodenalis

**Figure 1.**
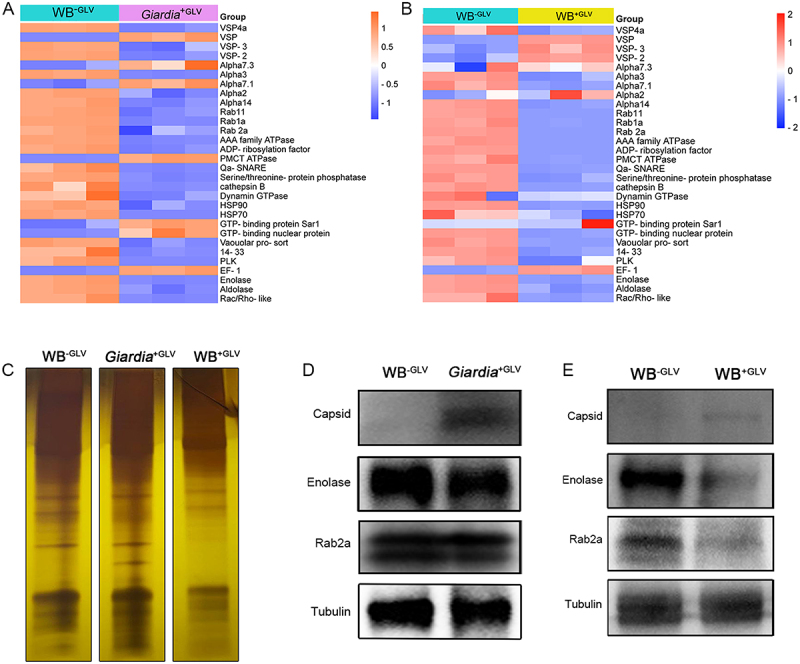
GLV infection modulates the levels of mRNA and protein in *G. duodenalis*. (A-B) Heatmap representations showing the mRNA abundance of vesicle biogenesis-related and virulence-associated genes in WB^−GLV^ *vs. Giardia*^+GLV^ and WB^−GLV^ *vs*. WB^+GLV^. (C) Silver-stained SDS-PAGE showing distinct protein patterns among WB^−GLV^, *Giardia*^+GLV^ and WB^+GLV^ trophozoites. (D-E) Western blot analysis of protein abundance in WB^−GLV^ *vs. Giardia*^+GLV^ and WB^−GLV^ *vs*. WB^+GLV^.

To elucidate the impact of Giardiavirus (GLV) infection on *Giardia*, we analyzed the mRNA abundance of vesicle biogenesis-related and virulence-associated genes among three groups of trophozoites: equal numbers of WB^−GLV^ (GLV-free), *Giardia*^+GLV^ (naturally GLV-containing), and WB^+GLV^ (WB^−GLV^ strain infected with GLV in vitro) using qPCR. In *Giardia*^+GLV^ isolates, the mRNA levels of most genes were decreased compared to WB^−GLV^ isolates, with the exception of GTP-binding nuclear protein, PMCT ATPase, VSP, Alpha7.3, and EF-1 (Figure 1A). To exclude potential variability caused by genetic differences among isolates, the same set of genes was analyzed in WB^+GLV^ strains. Similarly, most genes showed reduced mRNA levels in WB^+GLV^ strains compared to WB^−GLV^ isolates, except for EF-1, VSP, VSP-2, VSP-3, and Alpha2 (Figure 1B). To determine whether GLV infection also affects protein expression, we compared total protein profiles of equal numbers of WB^−GLV^, *Giardia*^+GLV^, and WB^+GLV^ trophozoites. Silver staining revealed distinct global protein expression patterns among the three groups, suggesting that GLV infection alters the overall proteome of *Giardia* (Figure 1C). Western blot analysis further confirmed these findings. Protein levels of Enolase and Rab2a were significantly decreased in *Giardia*^+GLV^ isolates compared to WB^−GLV^ isolates (Figure 1D, Supplementary Fig. S1A). In addition, the expression levels of Enolase and Rab2a were also downregulated in WB^+GLV^ strains compared to WB^−GLV^ isolates (Figure 1E, Supplementary Fig. S1B). Together, these results indicate that GLV infection remodels mRNA levels and protein abundance in *Giardia*.

### Giardiavirus infection enhances mRNA translation efficiency

**Figure 2.**
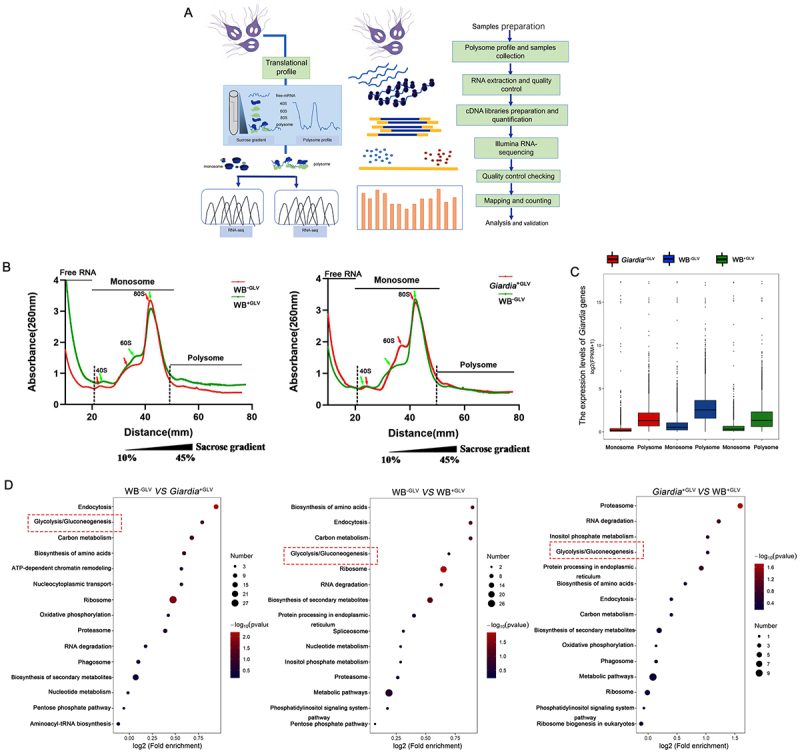
GLV infection enhances mRNA translation efficiency in *Giardia.* (A) Overview of polysome profiling and polysome-RNA-seq analyses performed on the WB^−GLV^, *Giardia*^+GLV^, and WB^+GLV^ trophozoites. (B) Polysome profiles recorded by ultraviolet absorbance at 260 nm, showing monosome (single ribosome – bound mRNA) and polysome (multiple ribosome – bound mRNA) fractions in WB^−GLV^, *Giardia*^+GLV^, and WB^+GLV^ trophozoites. The x-axis represents sucrose density gradients, the y-axis indicates OD260 absorbance. (C) FPKM analysis showing differences in gene expression levels in the fraction of monosome and polysome. (D) KEGG pathway enrichment analysis of genes with increased translation efficiency in WB^−GLV^ *vs.* *Giardia*^+GLV^, WB^−GLV^ *vs.* WB^+GLV^, and *Giardia*^+GLV^ *vs.* WB^+GLV^.

To investigate the effect of GLV infection on the mRNA translation efficiency in *Giardia*, polysome profiling was performed on WB^−GLV^, *Giardia*^+GLV^ and WB^+GLV^ trophozoites by recording the ultraviolet absorbance at 260 nm (Figure 2A). Translation efficiency was higher in the WB^+GLV^ strain compared to that of *Giardia*^+GLV^ and WB^−GLV^, as evidenced by a higher polysome/monosome ratio in the WB^+GLV^ strain (Figure 2B, Supplementary Fig. S2), indicating that GLV infection enhances mRNA translation efficiency. Then, polysome-RNA-seq analysis of monosomes and polysomes was performed for all three *Giardia* strains. The results showed that the expression levels of overall genes in the WB^−GLV^ isolate were higher in the monosome and polysome fractions, compared to those in the *Giardia*^+GLV^ and the WB^+GLV^ isolate (Figure 2C). This observation is consistent with the decreased gene abundance observed in GLV-infected *Giardia*, as shown in Figure 1A,B. These findings indicate that GLV infection rewires the host translation, including the enhancement of mRNA translation efficiency and the reduction of overall gene abundance.

KEGG pathway enrichment analysis of genes with upregulated translation efficiency provides insights into the biological processes modulated by GLV infection. In the WB^−GLV^ *vs. Giardia*^+GLV^ comparison, the top four enriched pathways were endocytosis, glycolysis/gluconeogenesis, carbon metabolism, and biosynthesis of amino acids. In the WB^−GLV^ *vs*. WB^+GLV^ comparison, the top four enriched pathways were biosynthesis of amino acids, endocytosis, carbon metabolism, and glycolysis/gluconeogenesis. In the *Giardia*^+GLV^ *vs*. WB^+GLV^ comparison, the top four enriched pathways were proteasome, RNA degradation, inositol phosphate metabolism, and glycolysis/gluconeogenesis. Notably, glycolysis/gluconeogenesis was consistently enriched across all three comparisons (Figure 2D), suggesting a robust association between GLV infection and regulation of the glycolytic pathway.

### Giardiavirus infection triggers energy metabolic reprogramming

**Figure 3.**
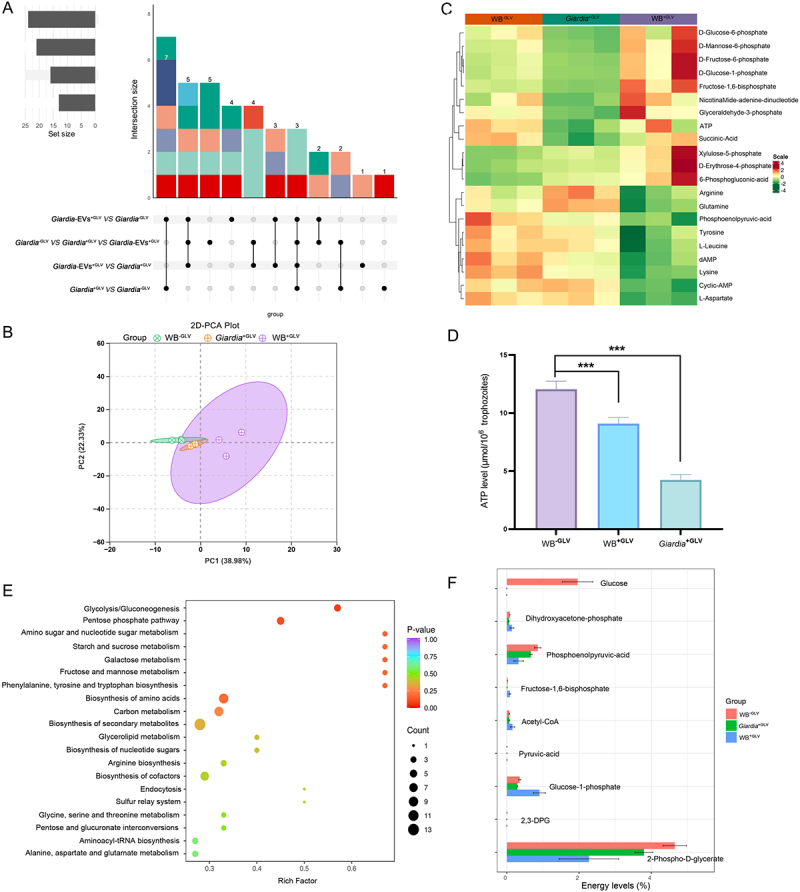
GLV infection induces energy metabolic reprogramming in *Giardia.* (A) Upset plot showing common and unique differential metabolites across comparisons. (B) Two-dimensional principal component analysis (PCA-2D) illustrating metabolic group separation and intragroup variability. (C) Heatmap hierarchical clustering of differential metabolite profiles in WB^−GLV^, *Giardia*^+GLV^, and WB^+GLV^ trophozoites. (D) Intracellular ATP content in three *Giardia* groups measured by multifunctional microplate reader. (E) KEGG pathway enrichment analysis of significantly differential metabolites in GLV-infected *Giardia*. (F) Energy distribution analysis of differential metabolites in WB^−GLV^, *Giardia*^+GLV^, and WB^+GLV^ trophozoites. Three independent experiments were performed. The data are presented as means ± SEM. ***P < 0.001.

Viruses lack self-sufficient metabolic networks and thus rely on host cellular energy to support their replication, with viral reproduction rates being regulated by host metabolic activity [35,36]. To directly assess whether GLV infection modulates energy metabolism in *Giardia*, metabolic profiling was performed on WB^−GLV^, *Giardia*^+GLV^ and WB^+GLV^ trophozoites. Our findings showed that, compared with WB^−GLV^, 13 and 24 differential energy metabolites were identified in *Giardia*^+GLV^ and WB^+GLV^, respectively. Additionally, 16 differential metabolites were detected in *Giardia*^+GLV^ compared with WB^+GLV^ (Figure 3A). Principal component analysis (PCA) revealed metabolic distinctions among the three *Giardia* groups, along with intra-group variability (Figure 3B). Heatmap hierarchical clustering and violin plots showed distinct metabolite profiles in three *Giardia* groups, with the levels of 21 metabolites significantly altered (Figure 3C, Supplementary Fig. S3). ATP levels in WB^−GLV^, *Giardia*^+GLV^ and WB^+GLV^ trophozoites were measured using an ATP assay kit. The results showed that ATP levels were reduced in the GLV-infected trophozoites (*Giardia*^+GLV^ and WB^+GLV^) compared to the GLV-free *Giardia* (WB^−GLV^), consistent with the metabolite profile shown in Figure 3C (Figure 3D). KEGG pathway enrichment analysis of the differential energy metabolites in GLV-infected trophozoites indicated that glycolysis/gluconeogenesis was the most significantly enriched pathway (Figure 3E). Furthermore, energy composition analysis also showed that the majority of altered metabolites were predominantly associated with glycolytic processes (Figure 3F). Taken together, these findings demonstrate that GLV infection reprograms energy metabolism in *Giardia*, and highlight a strong association between GLV infection and the glycolysis/gluconeogenesis pathway.

### GLV infection enhances glycolysis in *Giardia*

**Figure 4.**
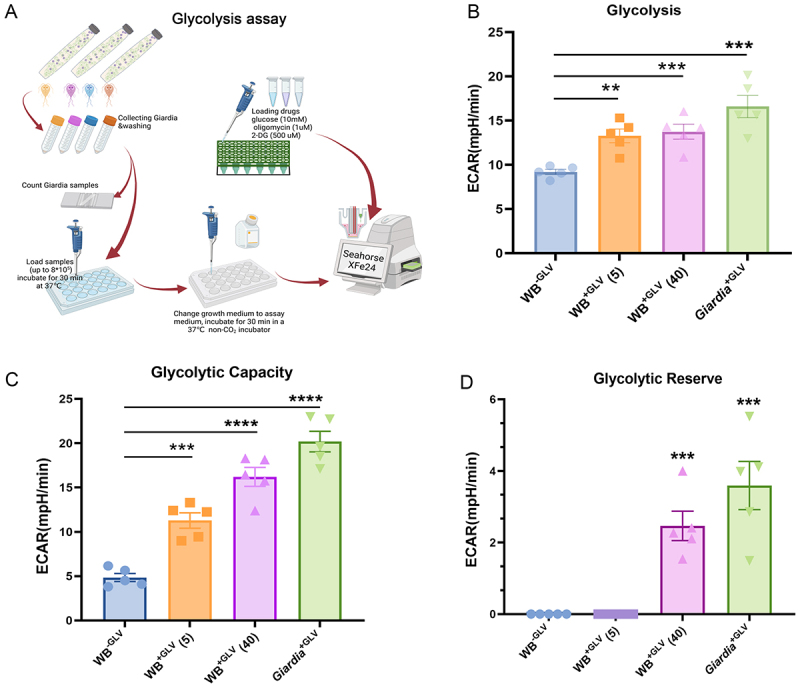
Measurement of glycolysis by the Seahorse XFe24 Flux Analyzer. (A) Schematic overview of the glycolysis stress assay. (B-D) Quantification of glycolysis (B), glycolysis capacity (C), and glycolysis reserve (D) using the Seahorse XFe24 Flux Analyzer. Three independent experiments were performed. The data are presented as means ± SEM. ** *P* < 0.01, ***P < 0.001, and ****P < 0.0001.

Glycolytic activity was assessed using the glycolytic stress assay with the Seahorse XF Analyzer [37]. To ascertain whether GLV infection affects the glycolytic pathway, glycolytic stress tests were performed on equal numbers of *Giardia* trophozoites. The WB^+GLV^ strain (from the 5th and 40th passages of GLV infection) was used as the experimental group, WB^−GLV^ and *Giardia*^+GLV^ isolates served as controls to evaluate the impact of GLV infection on glycolysis (Figure 4A). The results demonstrated that glycolysis was significantly elevated in the GLV-infected *Giardia* (WB^+GLV^ and *Giardia*^+GLV^), with a further increase observed in the 40th passage of GLV infection. This was evidenced by significant increases in glycolysis, glycolytic capacity, and glycolytic reserve (Figures 4B-D). Notably, there was no significant difference in GLV load between WB^+GLV^ trophozoites at the 5th and 40th passages (Supplementary Fig.S 4). These findings suggest that GLV infection promotes glycolysis in *Giardia* trophozoites, and that prolonged GLV passage further augments glycolytic activity.

### Distribution of the glycolytic enzyme Gd-enolase in *Giardia* trophozoites

**Figure 5.**
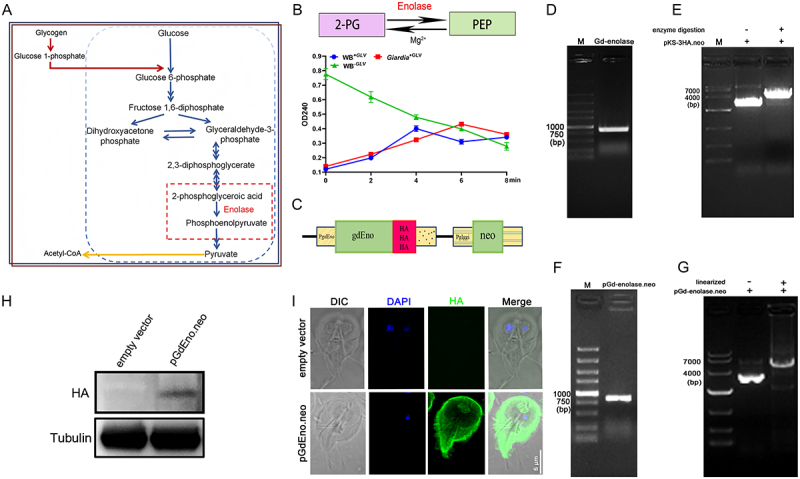
Intracellular distribution of Gd-enolase in *Giardia* trophozoites. (A) Overview of the glycolysis pathway. (B) Enolase activity was measured by monitoring the conversion of 2-phosphoglycerate (2-PGA) to phosphoenolpyruvate (PEP) at 240 nm. (C) Schematic representation of plasmid pGd-enolase.Neo. (D) Amplification of an 820-bp DNA fragment of the Gd-enolase gene from *Giardia* genomic DNA via PCR. (E) Cloning of *NotI* and *SalI* sites into plasmid pKS-3 HA.Neo to generate pGd-enolase.Neo. (F) Identification of pGd-enolase.Neo monoclonal colonies by colony PCR. (G) The plasmid of pGd-enolase.Neo was linearized. (H) The linearized pGd-enolase.Neo plasmid was electroporated into 1 × 10^7^ *Giardia* trophozoites. Expression of HA-tagged Gd-enolase was confirmed by western blot analysis. (I) Immunofluorescence of HA‑tagged Gd‑enolase and control (pKS‑3 HA.Neo) in the WB^+GLV^ trophozoites. Scale bar, 5 μm. Observation of the resulting transfectants was made in at least three independent experiments.

Given that GLV infection enhances glycolysis in *Giardia*, and energy distribution analysis (Figure 3F) revealed a relatively high proportion of glycolytic metabolites—2-phosphoglycerate (2-PGA) and phosphoenolpyruvate (PEP), we next investigated enolase, the enzyme that catalyzes the interconversion of these two metabolites (Figure 5A). PEP exhibits a characteristic UV absorbance at 240 nm, and changes in absorbance at this wavelength reflect PEP formation. Under identical 2-PG doses, PEP formation profiles were compared among different groups to assess enolase catalytic activity across *Giardia* lysates.The results showed that Gd-enolase activity in GLV-infected *Giardia* (*Giardia*^+GLV^ and WB^+GLV^) was distinct from that in the GLV-free *Giardia* (WB^−GLV^) (Figure 5B), suggesting that Gd-enolase is involved in GLV-driven glycolysis in *Giardia*. To facilitate subsequent investigation of Gd-enolase function, transgenic *Giardia* trophozoites expressing HA-tagged Gd-enolase were constructed (Figure 5C). An 820 bp fragment of the Gd-enolase gene was successfully amplified and cloned into the *NotI*/*SalI*-digested pKS-3 HA.neo vector (Figures 5D-E). The recombinant pKS-enolase.neo plasmid, containing the HA tag, was verified and linearized (Figures 5F-G), and subsequently introduced into the WB^+GLV^ strain via electroporation. Western blot analysis of the resulting WB^+GLV^ strain extracts confirmed the expression of HA-tagged Gd-enolase as an immunoreactive band with a molecular weight of 49 kDa. In contrast, the WB^+GLV^ strain carrying the vector control, pKS-3 HA.neo, did not produce any immunoreactive bands in the same analysis (Figure 5H). Immunofluorescence analysis showed that Gd-enolase was predominantly localized in the cytoplasm and along the plasma membrane of WB^+GLV^ trophozoites (Figure 5I), indicating that HA-tagged WB^+GLV^ trophozoites resistant to neomycin were successfully obtained.

### *G.* *duodenalis* enolase regulates GLV replication

**Figure 6.**
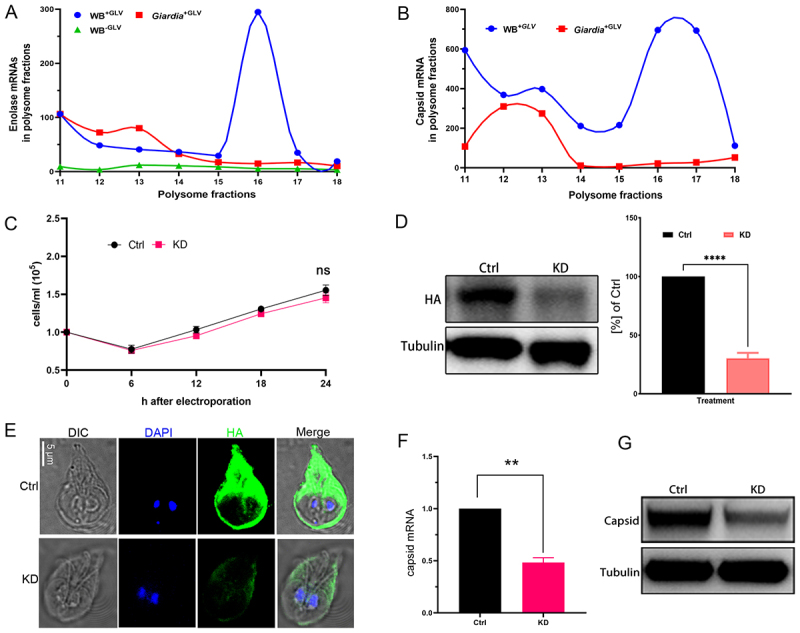
GLV employs *G. duodenalis* enolase to facilitate its replication. (A-B) qPCR analysis of Gd‑enolase mRNA (A) and GLV capsid mRNA (B) across polysome fractions in *Giardia*. (C) Growth curves of control and Gd-enolase-depleted cell cultures were observed and analyzed using light microscopy. (D) Western blot showing Gd-enolase-HA levels after 24 h of morpholino treatment. (E) Immunofluorescence staining of control and Gd-enolase-depleted cells. Scale bar, 5 μm. (F-G) qPCR (F) and Western blot (G) analyses showing the levels of GLV capsid mRNA and protein at 24 h post‑knockdown. All data presented are representative of three independent experiments. **P ≤ 0.01, ***P ≤ 0.001, and ****P < 0.0001.

Enolase, a glycolytic enzyme, is recognized as a “moonlighting protein” owing to its multiple cellular functions beyond metabolism. In addition to its role in energy production, enolase has been implicated in the replication of various viruses, including HIV, Sendai virus, and dengue virus [38–41]. Therefore, we investigated whether Gd-enolase plays a role in regulating GLV replication in *Giardia*. To further explore this potential relationship, the distribution of enolase mRNA across polysome fractions was analyzed in WB^−GLV^, *Giardia*^+GLV^ and WB^+GLV^ trophozoites, and the distribution of GLV capsid mRNA was assessed in *Giardia*^+GLV^ and WB^+GLV^ trophozoites. qPCR analysis revealed that enolase mRNA in the WB^+GLV^ trophozoites predominantly accumulated in polysome fractions 15–17, whereas in *Giardia*^+GLV^ trophozoites, it was mainly present in fractions 11–14. In contrast, no significant accumulation of enolase mRNA was observed in any polysome fractions from WB^−GLV^ trophozoites (Figure 6A). A similar distribution pattern was observed for GLV capsid mRNA, which was accumulated in fractions 15–17 in WB^+GLV^ and in fractions 11–14 in *Giardia*^+GLV^ (Figure 6B). In comparison, other genes such as Alpha-6 giardin, 14–3-3, and Rab2a did not show polysome distribution patterns consistent with that of the GLV capsid mRNA (Supplementary Fig. S5), further supporting the potential role of Gd-enolase in regulating GLV infection.

To further investigate the role of Gd-enolase in GLV infection, we employed an antisense translation-blocking morpholino to specifically deplete Gd-enolase expression in WB^+GLV^ trophozoites. After 24 h of culture, the growth rate of the knockdown group was monitored. The results showed that there was no significant difference between the knockdown group and the nonspecific morpholino control (Figure 6C). Western blot analysis of the HA-tag demonstrated a marked reduction in the immunoreactive band in the knockdown group (Figure 6D). This finding was further corroborated by immunofluorescence analysis, which showed a marked decrease in HA-tagged fluorescence intensity in the knockdown group compared to the control (Figure 6E), confirming successful suppression of Gd-enolase expression. We then measured GLV capsid mRNA and protein levels in both control and knockdown groups using qPCR and western blot analysis. The results showed that depletion of Gd-enolase significantly reduced GLV capsid expression at both the mRNA and protein levels (Figures 6F–G), suggesting that Gd-enolase positively regulates GLV replication in *Giardia*.

## Discussion

Viruses are obligate intracellular parasites that rely entirely on host cellular machinery for their survival and propagation [42,43]. However, our understanding of how protozoan viruses influence the physiology of their host parasite remains limited. Inspired by previous studies showing that *Leishmania* RNA virus 1 (LRV1) can modulate protein abundance and translational efficiency in *Leishmania* [18], we investigated whether *Giardiavirus* (GLV) exerts similar regulatory effects in *Giardia duodenalis*. Previous studies have shown that GLV is present within extracellular vesicles and modulates the pathogenicity of *Giardia* in murine models [8,10,44]. Additionally, our unpublished data indicate that GLV infection affects the quantity of extracellular vesicles released by *Giardia*. Therefore, we selected 30 genes associated with vesicle biogenesis and the virulence of *Giardia* for qPCR analysis. Our results found that GLV infection led to a reduction in both mRNA and protein levels. Viruses can hijack the host’s translational machinery to preferentially promote the synthesis of viral proteins [45]. Translation efficiency is typically defined as the number of proteins synthesized per unit time by per mRNA, reflecting the combined effects of translation initiation and elongation efficiencies. However, as translation initiation is typically the primary rate-limiting step in the translation process, translation efficiency is often approximated by translation initiation efficiency [46,47]. Next, polysome profiling and RNA-seq analyses observed that GLV infection enhances mRNA translation efficiency in *Giardia*. Intriguingly, despite this increase in translational efficiency, the levels of host proteins decreased following GLV infection. Previous studies have reported that a reduction in mRNA and protein abundance does not necessarily indicate decreased translational efficiency, as multiple viral mechanisms – such as inhibition of translation initiation, ribosome redistribution, and selective translation mediated by IRES elements or uORFs – can sustain or even enhance translation efficiency despite global mRNA reduction [48–50]. Similar mechanisms have been documented in viruses such as influenza virus and respiratory syncytial virus (RSV) [49,51], and may likewise underlie the increased translation efficiency observed during GLV infection in *Giardia*. Furthermore, the presence of IRES elements within GLV mRNAs supports the possibility that GLV exploits host translation machinery in a manner analogous to other RNA viruses [52]. Additionally, we hypothesize that GLV infection induces ribosome redistribution within *Giardia*, with viral mRNAs potentially competing with host mRNAs for ribosome access, leading to a reduction in host protein synthesis. As a compensatory mechanism, the translation efficiency of *Giardia* mRNAs may be relatively enhanced to ensure the production of essential proteins for survival. However, this mechanism requires further investigation.

Since KEGG pathway enrichment analysis of polysome-RNA-seq data revealed a potential association between GLV infection and the glycolysis/gluconeogenesis pathway, we investigated whether GLV infection alters the metabolic profile of *Giardia*. Viruses rely on host cell resources for energy and metabolite precursors necessary to produce progeny viruses, and have evolved multiple mechanisms to remodel host metabolic systems for optimal infection [53–55]. To evaluate metabolic alterations, we performed targeted metabolomic profiling on equal numbers of GLV-free trophozoites (WB^−GLV^) and GLV-infected trophozoites (*Giardia*^+GLV^ and WB^+GLV^). Principal component analysis (PCA) was applied to the metabolic datasets to obtain an overview of global metabolic differences among the three groups and to assess the degree of variability within each group [56]. Our results found that notable intragroup variation in metabolite profiles within the WB^+GLV^ group, as shown in the PCA-2D plots, suggesting that GLV infection may contribute to metabolic heterogeneity among *Giardia*. Additionally, a total of 21 significantly altered metabolites were identified across the three groups. ATP levels were reduced in GLV-infected *Giardia* strains. Similar phenomena have been observed in other viral infections, such as white spot syndrome virus (WSSV) and classical swine fever virus (CSFV). Notably, WSSV infection upregulates glycolysis while suppressing oxidative phosphorylation, leading to decreased ATP production [57,58]. Whether GLV infection in *Giardia* follows a similar mechanism – enhancing glycolysis while downregulating oxidative phosphorylation – remains to be elucidated. KEGG analysis showed that glycolysis/gluconeogenesis was the most prominently enriched pathways. The Seahorse analyzer is a powerful tool for measuring cellular glycolytic activity, and has been increasingly applied to parasite research, including *Toxoplasma gondii* and *Giardia* [30,59]. We employed this platform to further investigate glycolysis in *Giardia* trophozoites. Our results demonstrate that GLV infection significantly enhances glycolysis in *Giardia* in a manner dependent on viral passage rather than viral load. Collectively, our data show that GLV infection induces energy metabolism reprogramming in *Giardia*, with a pronounced shift toward glycolysis. These findings provide foundational insights into the metabolic pathways involved in GLV-*Giardia* interactions.

Viruses are known to manipulate host proteins, which can function as either positive or negative regulators of viral infection [60]. For example, during white spot syndrome virus (WSSV) infection in shrimp, the virus enhances glycolysis by interacting with the rate-limiting glycolytic enzyme triosephosphate isomerase (TPI), thereby increasing its enzymatic activity and facilitating viral replication [57]. Enolase, a metal ion-activated enzyme involved in glycolysis, plays a vital role in viral replication [60–62]. Previous studies have shown that *Giardia* enolase is involved in the glycolytic pathway and is abundantly expressed in the cytoplasm of the parasite [63]. In this study, energy distribution analysis revealed two glycolytic intermediates, 2-phosphoglycerate (2-PGA) and phosphoenolpyruvate (PEP), were present at relatively high levels in three *Giardia* groups. Enolase catalyzes the reversible conversion of 2-PGA to PEP within the glycolysis pathway [64]. In Figure 5B, PEP formation was measured under identical 2-PG concentrations across different groups to compare enolase catalytic activity in *Giardia* lysates. We speculate that the initially higher OD values at 240 nm observed in WB^−GLV^ compared to *Giardia*^+GLV^ and WB^+GLV^ may be attributed to the higher enolase expression levels detected in WB^−GLV^, which gradually declined as 2-PG was consumed during the reaction.

Enolase plays a pivotal role in viral replication. In HBV infection, enolase expression is upregulated, and its silencing significantly inhibits viral replication [62]. HIV infection downregulates enolase during latency and upregulates it during the active replication phase [60,65]. To investigate its role in GLV replication, we performed morpholino-mediated knockdown of Gd-enolase, a widely used and technically mature method for gene function studies in *Giardia* [66]. Our results showed that inhibition of Gd-enolase translation led to a reduction in GLV load. Interestingly, GLV-infected *Giardia* (*Giardia*^+GLV^ and WB^+GLV^) exhibited lower enolase levels compared to the WB^−GLV^, seemingly contradicting the knockdown results. We still speculate that this contradiction may be explained by the presence of IRES in GLV mRNAs, which enable the virus to hijack host ribosomes for preferential translation of viral proteins, thereby nonspecifically suppressing host protein synthesis, including that of enolase [52]. Research on GLV mechanisms remains scarce, and available information is extremely limited. While this study does not fully resolve all mechanistic questions, future investigations will aim to clarify these aspects in greater detail. Looking forward, our previous findings that GLV infection attenuates *Giardia*-induced intestinal pathology and mitigates growth impairment in mice [10], elucidating the regulatory effects of GLV on the physiology of *Giardia* may provide novel targets for the development of vaccines and therapeutics against giardiasis. Similar strategies have been demonstrated in leishmaniasis, where vaccination with the LRV1 capsid protein prevents disease exacerbation [67]. In addition, enzymes in parasite metabolic pathways have been investigated as potential drug targets in protozoan, such as *Trichomonas vaginalis* and *Plasmodium falciparum* [68]. *Giardia* enolase has been reported as a secreted moonlighting protein that induces necroptotic damage in IEC-6 cells through plasminogen activation and TNFα- and AIF-dependent pathways, and is therefore recognized as a virulence factor [69]. Collectively, these findings suggest enolase may act as a GLV-regulated virulence factor and could have potential as a therapeutic target for giardiasis.

## Conclusion

In summary, Giardiavirus (GLV) infection modulates the physiology of *G. duodenalis* by reducing mRNA and protein abundance, while concurrently enhancing mRNA translation efficiency and reprogramming host energy metabolism. Glycolysis/gluconeogenesis is the most significantly enriched metabolic pathway in GLV-infected *Giardia*. Moreover, GLV infection markedly enhances glycolysis in *Giardia*, with this effect intensifying over successive GLV passages. Notably, the glycolytic enzyme Gd-enolase plays an important role in regulating GLV replication within *Giardia* trophozoites. Given that GLV infection has been reported to attenuate the pathogenicity of *Giardia*, our findings not only deepen the understanding of GLV-*Giardia* interaction mechanisms but also suggest promising metabolic targets for the development of vaccines and therapeutic strategies against giardiasis.

## Supplementary Material

Supplementary Figure 2.tif

Supplementary Figure 4.tif

Supplementary Table 1.docx

Clean Copy of Supplementary figure legends- QVIR-2025-0493.R1.docx

Supplementary Table 3.docx

Supplementary Figure 5.tif

Supplementary Figure 1.tif

Supplementary Figure 3.tif

Supplementary Table 2.docx

## Data Availability

The data that support the findings of this study are openly available in Figshare at https://doi.org/10.6084/m9.figshare.29432195, reference number [70]. The metabolism data are available in the MetaboLights database under accession number MTBLS12615 (https://www.ebi.ac.uk/metabolights/MTBLS12615), reference number [71]. The RNA-seq datasets have been deposited in the NCBI Gene Expression Omnibus (GEO) under the accession number GSE269427 (https://www.ncbi.nlm.nih.gov/geo/query/acc.cgi?acc=GSE269427), reference number [72].
