# Supplementary figures and images for: Giardiavirus rewires host translation and glycolytic metabolism to support its replication in Giardia duodenalis
Source: Virulence. 2025 Dec 24;17(1):2605746. doi: 10.1080/21505594.2025.2605746 (PMC12758212; doi:10.1080/21505594.2025.2605746)

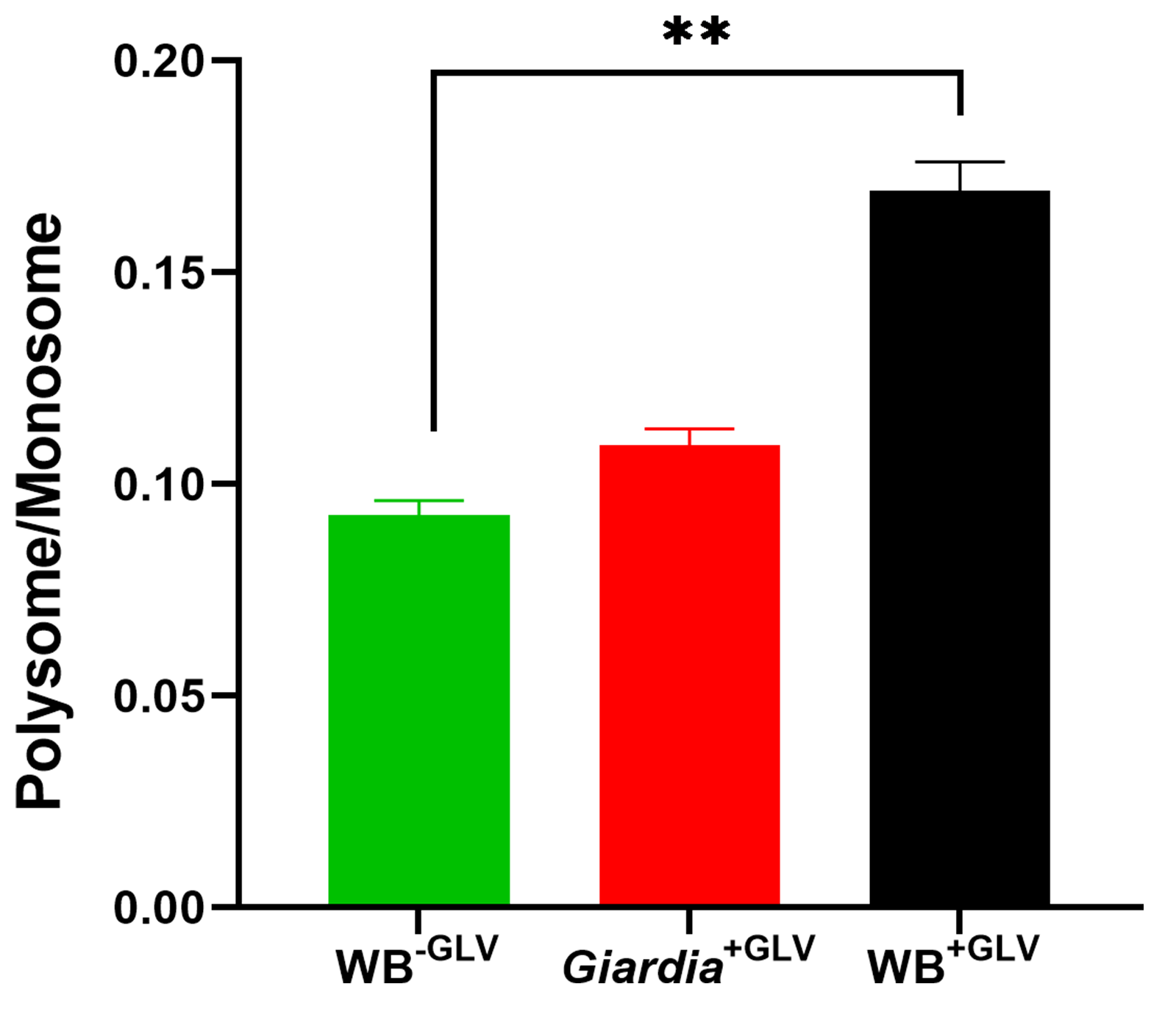

Supplement: Supplementary Figure 2.tif [file KVIR_A_2605746_SM6949.tif]

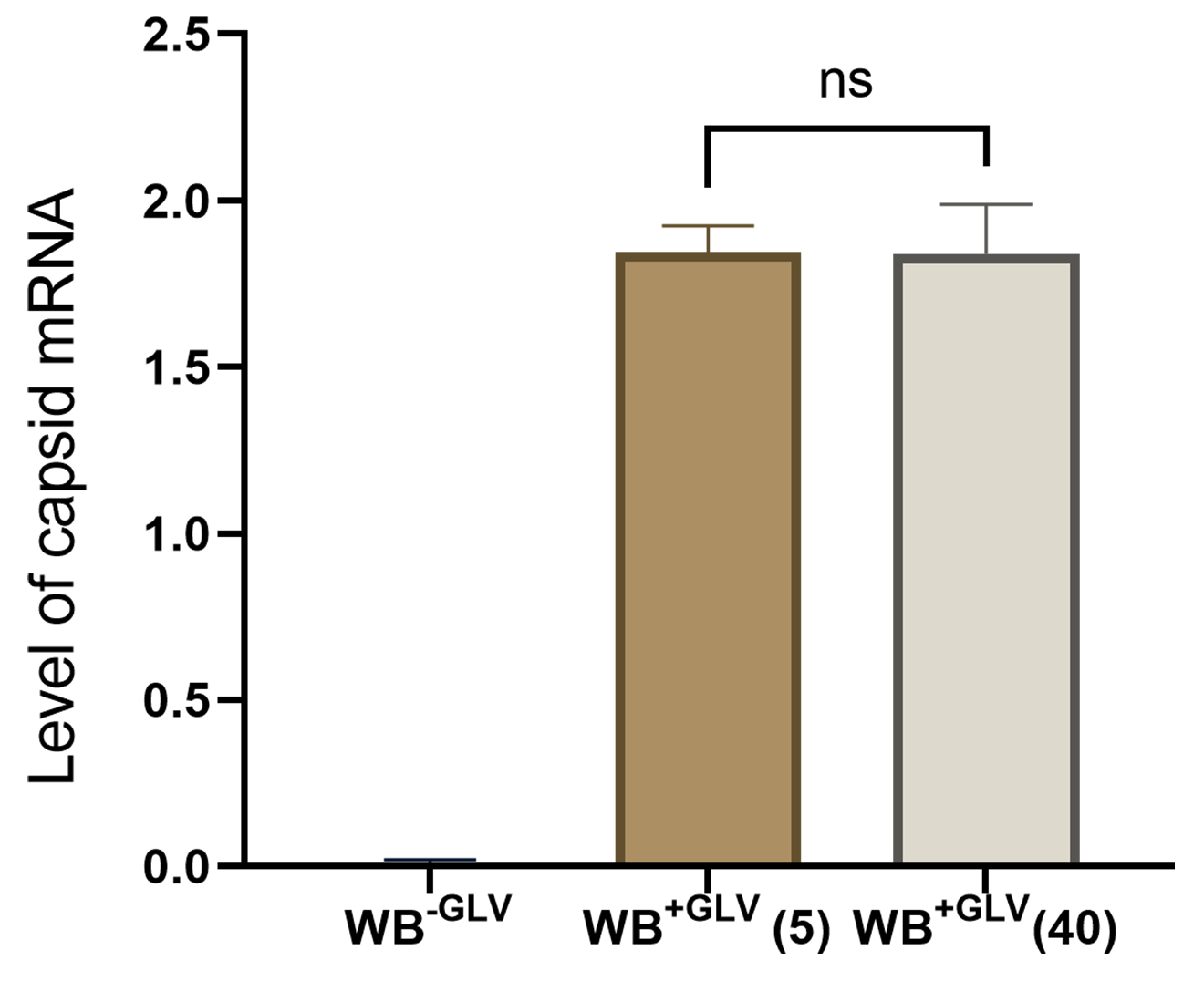

Supplement: Supplementary Figure 4.tif [file KVIR_A_2605746_SM6948.tif]

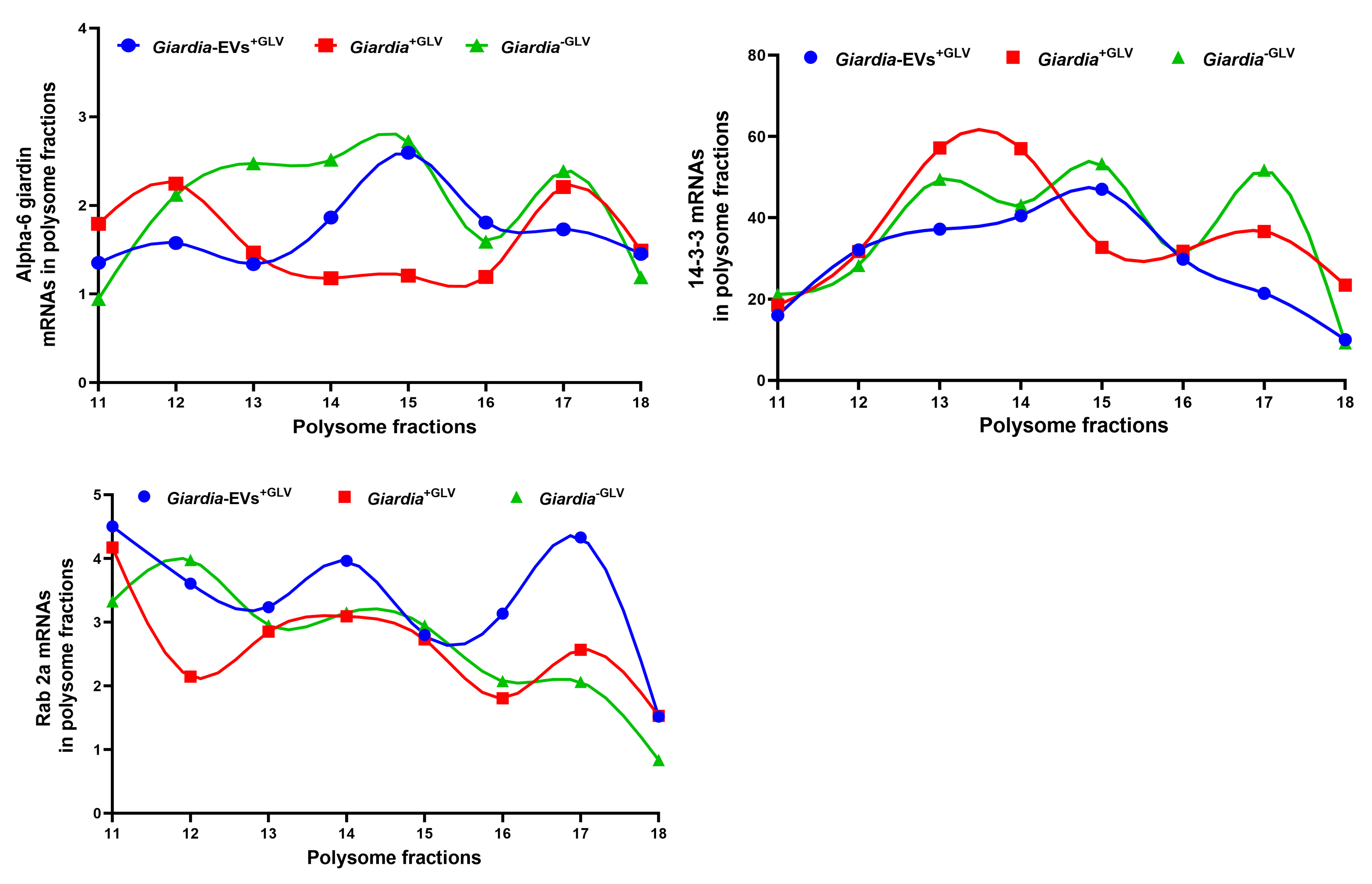

Supplement: Supplementary Figure 5.tif [file KVIR_A_2605746_SM6944.tif]

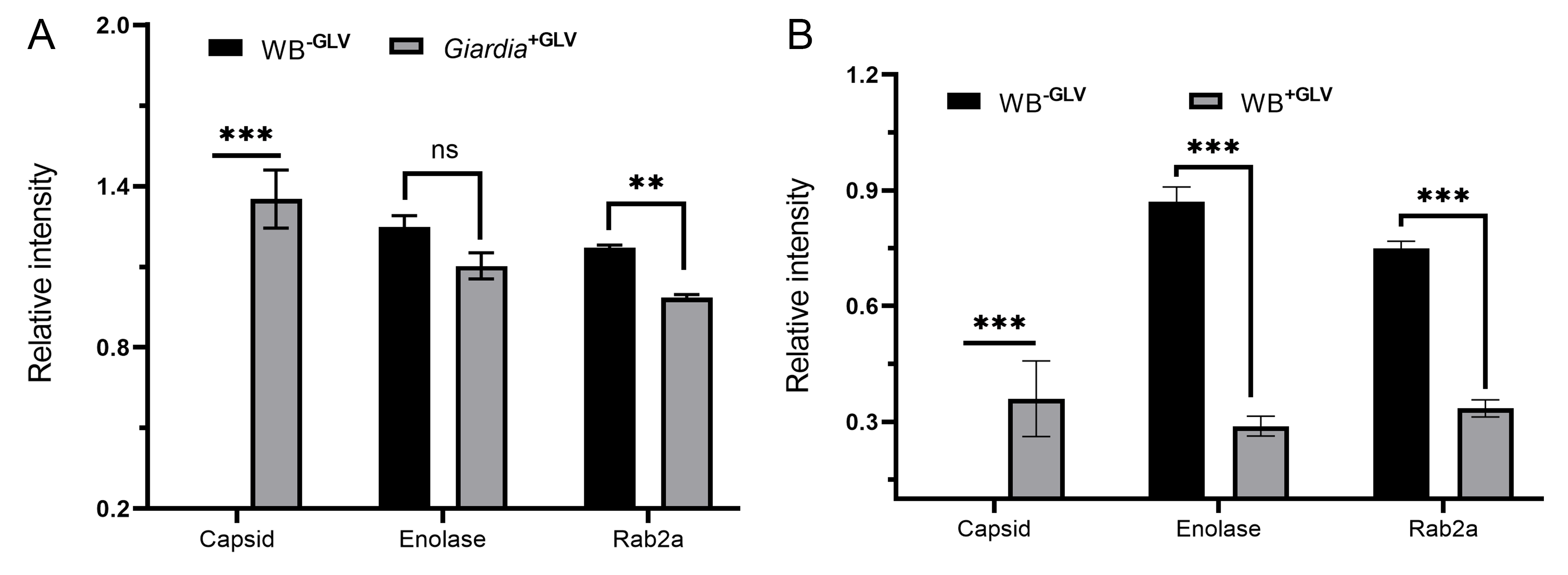

Supplement: Supplementary Figure 1.tif [file KVIR_A_2605746_SM6943.tif]

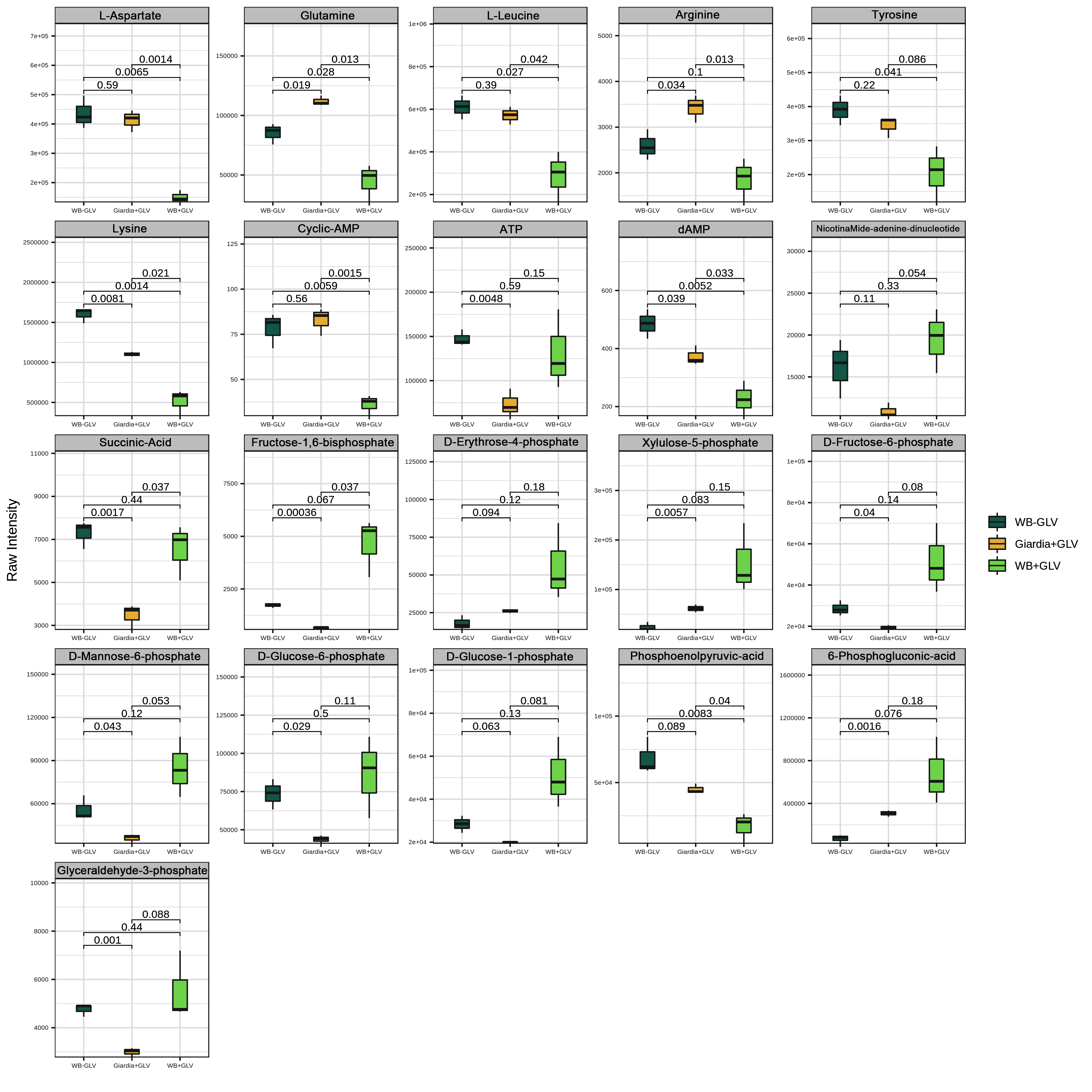

Supplement: Supplementary Figure 3.tif [file KVIR_A_2605746_SM6942.tif]
