# Supplementary material for: Giardiavirus rewires host translation and glycolytic metabolism to support its replication in Giardia duodenalis
Source: Virulence. 2025 Dec 24;17(1):2605746. doi: 10.1080/21505594.2025.2605746 (PMC12758212; doi:10.1080/21505594.2025.2605746)
Supplement: Supplementary Table 1.docx [file KVIR_A_2605746_SM6947.docx]

**Supplementary Table.1**

The 30 genes specific primers

| Primers name | Sequence (5′ to 3′) |
| --- | --- |
| Rab2a F | 5´-CTCGAAGAATGCCGCAC-3´ |
| Rab2a R | GCAATTATCATGAACGCCTC |
| Rab1a-F | AGGAGATTGCAAACAACGC |
| Rab1a-R | CATTGATGGAACCCGCACT |
| ADP-rf1-F | CACACTCTACTCGGTGAGG |
| ADP-rf1-R | GATGTACCAGTCGCGCTT |
| AAA family ATPase-F | GTGCGTCCTCTTCTTCGATG |
| AAA family ATPase-R | GTCCTTACGCACACCAACAC |
| HSP 70-F | AGCAAAGAACTACGCACCGAT |
| HSP 70-R | CGCCCATCCTTATTGATCACC |
| Dynamin GTPase-F | TTAATACCCTGCACCCCGACT |
| Dynamin GTPase-R | TCGCCAAAGCCCTGATTCG |
| Cathepsin B-F | CGCCTTCTTGGTCTACTCCG |
| Cathepsin B-R | CCGTAGCCCACCATCTCG |
| GTP-bNP RAN/TC4-F | GACAGTTAAGTTCCACCGCTC |
| GTP-bNP RAN/TC4-R | ATGCTCAGAATCGGAAGCTC |
| Qa-SNARE 1-F | CTACCAGCAGACCGTGGC |
| Qa-SNARE 1-R | CATCTGCTCCTCGGCCTTC |
| GTP-bp Sar1-F | GACTCCGCAGATCCATCGC |
| GTP-bp Sar1-R | ACGGCTGTCTGGATATCAACC |
| HSP 90-alpha-F | CTCATCGGGCAGTTCGGT |
| HSP 90-alpha-R | CCACTTGAAGCAGCTGTCG |
| 14-3-3-F | CTCTGCCCTCAACGCCTAC |
| 14-3-3-R | AAGTTCAGAGCCAGCCCGAG |
| PMCT ATPase 2-F | TGCTGCCATTCTCACGTTC |
| PMCT ATPase 2-R | GATCATATAGCCGACCCACT |
| Vacuolar protein sorting 35-F | TTCGTACAGCGCACAAC |
| Vacuolar protein sorting 35-R | CTTAAGCTCACGGATACTGTC |
| Rab11-F | CTACCGCTCGATCACGTC |
| Rab11-R | GCGTTTCTAAGCTCCGTCAAC |
| Serine/threonine-F | TCTCTGCCCACCATTGACG |
| Serine/threonine-R | TACGAGTGCTCCCACGTC |
| Alpha-7.3-F | ACCAGCTCTTCGGGGAC |
| Alpha-7.3-R | CCTCTGCATCACCCTGGAC |
| Alpha-2-F | CGAAGACTGGCACGAGAC |
| Alpha-2-R | AGTTTCTCTGCTTCCTCGTC |
| Alpha-3-F | GACTGGAACAATACCGTCGTT |
| Alpha-3-R | TCGCCATTGATGTTGTTCCT |
| VSP-F | AAGCATCTGACCCCGACG |
| VSP-R | TGCCGCCCTCATTAAGCT |
| VSP-2-F | TGCCAAACCTGTGCGAAC |
| VSP-2-R | ATTGGCTGCACTACACGTCT |
| VSP-3-F | CTACCAAACGAGTCAAGCACCT |
| VSP-3-R | TCCGCACGAAACAACCGAGT |
| Alpha-14-F | TCTGACGATAGAGACCCACG |
| Alpha-14-R | ATATACTTGTCCCCGCAGTC |
| Vps4a-F | CGGCGCTGAGAAGAAAGACT |
| Vps4a-R | CGCAACAGGCTACTCACAGT |
| Alpha-7.1-F | AGATCAAGGCGCTCATCGAC |
| Alpha-7.1-R | AGGTCCTTGTAGTCCCCGTTC |
| PLK-F | GTCACGTTTATGAGCGAGAA |
| PLK-R | CTATTCCCCTCCCTGACCGA |
| Enolase-F | AAGTACATCACAAAGGCCATCG |
| Enolase-R | TCCAGCCATAAGACATCGCAAG |
| Aldolase-F | TGGCGCATACAAATTCAAATCCG |
| Aldolase-R | CGCCATACTTGTTGATCATATCC |
| EF-1-F | GAAGCACATCAACCTCGTCGT |
| EF-1-R | TCCATGATGTTGTCCCCGGT |
| Rac/Rho-like-F | TACATATCAAAGCCGTAGTGGT |
| Rac/Rho-like-R | TCGCAATTTGTCGTAATCCTC |
| 18s-F | AAGACCGCCTCTGTCAATAA |
| 18s-R | GTTTACGGCCGGGAATACG |
| Giardiavirus capsid-F | ACTTCCTTTCGAGCTTTAACGTG |
| Giardiavirus -capsid-R | TGACAGTTTGGCTCGTGTCA |
| *Giardia* actin-F | CAGAACTGGCGTCAAACGTG |
| *Giardia* actin-R | TTTCCTCCATACCACACGGC |
