# Supplementary material for: Giardiavirus rewires host translation and glycolytic metabolism to support its replication in Giardia duodenalis
Source: Virulence. 2025 Dec 24;17(1):2605746. doi: 10.1080/21505594.2025.2605746 (PMC12758212; doi:10.1080/21505594.2025.2605746)
Supplement: Clean Copy of Supplementary figure legends- QVIR-2025-0493.R1.docx [file KVIR_A_2605746_SM6946.docx]

# Supplementary Materials

**Supplementary Fig. 1**

Densitometric analysis of Capsid, Enolase, and Rab2a expression levels
(A) Quantified relative protein levels corresponding to Figure 1D are displayed as bar graphs. (B) Quantified relative protein levels corresponding to Figure 1E are displayed as bar graphs.

**Supplementary Fi. 2**

Translation efficiency of WB^-GLV^, *Giardia*^+GLV^ and WB^+GLV^ trophozoites

Polysome profiling was performed on WB^-GLV^, *Giardia*^+GLV^ and WB^+GLV^ trophozoites by

recording the ultraviolet absorbance at 260 nm. Translation efficiency was assessed by

calculating the ratio of the area under the curve (AUC) for polysome fractions to that of

monosome fractions.

**Supplementary Fig. 3**

Violin plots of 21 differential metabolites in WB^-GLV^, *Giardia*^+GLV^ and WB^+GLV^ trophozoites.

**Supplementary Fig. 4**

qPCR analysis of GLV load in WB^+GLV^ trophozoites at the 5th and 40th passages.

**Supplementary Fig. 5**

Distribution of Alpha-6 giardin, 14-3-3, and Rab2a mRNA across polysome fractions in WB^-GLV^, *Giardia*^+GLV^ and WB^+GLV^ trophozoites analyzed by qPCR.
