# Supplementary material for: Giardiavirus rewires host translation and glycolytic metabolism to support its replication in Giardia duodenalis
Source: Virulence. 2025 Dec 24;17(1):2605746. doi: 10.1080/21505594.2025.2605746 (PMC12758212; doi:10.1080/21505594.2025.2605746)
Supplement: Supplementary Table 3.docx [file KVIR_A_2605746_SM6945.docx]

**Supplementary Table.3**

Morpholine sequence

| Gene | Accession number | Morpholino sequence (5′ to 3′) |
| --- | --- | --- |
| Standard control | NA | CCTCTTACCTCAGTTACAATTTATA |
| Enolase | GL50803_0011118 | CCTTGATCGTAGACGGAGCCTCCAT |
