# Supplementary material for: Giardiavirus rewires host translation and glycolytic metabolism to support its replication in Giardia duodenalis
Source: Virulence. 2025 Dec 24;17(1):2605746. doi: 10.1080/21505594.2025.2605746 (PMC12758212; doi:10.1080/21505594.2025.2605746)
Supplement: Supplementary Table 2.docx [file KVIR_A_2605746_SM6941.docx]

**Supplementary Table.2**

Construction of the plasmid pKS-enolase.neo

| Gd-enolase-F | CTTGGGCCCCAGGAGTACAT |
| --- | --- |
| Gd-enolase-R | CTTATGGTTTCGAGGCCTGGAAG |
| pGd.enolase.neo infusion-F | agctccaccgcggtggcggccgcCTTGGGCCCCAGGAGTACA |
| pGd.enolase.neo infusion-R | ttccttaagctcgaggtcgacCTTCCAGGCCTCGAAACCA |
| pGd.enolase.neo-linearized-F | ACCTTGGCTGGTGCAGACG |
| pGd.enolase.neo-linearized-R | AGATGTTTGCGAGCTCAGCG |
